# Is two cuter than one? number and relationship effects on the feeling of *kawaii* toward social robots

**DOI:** 10.1371/journal.pone.0290433

**Published:** 2023-10-18

**Authors:** Masahiro Shiomi, Rina Hayashi, Hiroshi Nittono

**Affiliations:** 1 Interaction Science Laboratory, Advanced Telecommunications Research Institute International, Kyoto, Japan; 2 Graduate School of Engineering, Osaka University, Suita, Japan; 3 Graduate School of Human Science, Osaka University, Suita, Japan; SGH Warsaw School of Economics: Szkola Glowna Handlowa w Warszawie, POLAND

## Abstract

*Kawaii*, which is a Japanese word that means cute, lovely, and adorable, is an essential factor in promoting positive emotions in people. The characteristics of a target’s appearance that induce such feelings of *kawaii* have been thoroughly investigated around the notion of Konrad Lorenz’s famous baby schema. Such knowledge has been exploited to design the appearance of commercial products to increase their social acceptance and commercial appeal. However, the effects of the number of targets and showing their mutual relationships (like friendship) have not been investigated in the context of *kawaii*. Therefore, in this study, we conducted three web-based experiments and focused on how such factors contribute to feelings of *kawaii* toward social robots. In Experiment 1, the feelings of *kawaii* toward static images of targets were compared when they appeared alone or with another target: persons (twin boys/girls), non-human objects (cherries), and social robots. The results showed that the feeling of *kawaii* was stronger for two targets that displayed a mutual relationship (e.g., looking at each other and/or making physical contact) than for one target alone and for two-independent targets. In Experiment 2, these findings were replicated using video clips of robots. Two-related targets were rated as more *kawaii* than two-independent targets or a single target. These two experiments consistently show the advantage of multiple robots that display their mutual relationship for enhancing the viewer’s feeling of *kawaii*. Experiment 3 examined the effect of the number of robots (from one to ten) and found that two robots induced the strongest feeling of *kawaii*. These results indicate that not only the physical characteristics of a target itself but also the number of targets and their perceived relationships affect feelings of *kawaii*.

## Introduction

Social robots that interact with people in daily environments have become popular worldwide, such as Paro [1], AIBO [2], and LOVOT [3]. A common design policy of these robots is the Japanese word *kawaii*, which has become a very popular word [4,5]. This adjective, whose English meaning denotes cute, lovely, or adorable, also expresses one’s affective feeling induced by the perception of objects imbued with such characteristics [5,6]. The feeling of *kawaii* is related not only to positive emotions but also to such behavioral changes as smiling and caretaking [5,7–10]. One elicitor of the feeling of *kawaii* is ethnologist Konrad Lorenz’s baby schema, which has been known to induce perceptions of cuteness [11,12]. The baby schema is a set of facial and bodily features typically possessed by young animals and babies, including a relatively large head and big eyes. Researchers employed a baby schema as a design policy to investigate its effects on such machines as cars and robots [13–16].

Are only appearances essential to improve feelings of *kawaii*? From a commercial aspect, the effort to change the appearance of mass-produced consumer products, including social robots, seems to eventually reach a saturation point. If different factors other than appearances can increase feelings of *kawaii*, such approaches might be worth exploring. Past studies have suggested two approaches for this purpose: designing specific patterns of behavior and increasing the number of targets. For the former, a few studies used robots and investigated what kinds of behaviors imparted *kawaii* feelings to people, e.g., a mobile robot’s locomotion behavior design [17] and a touch behavior design of both a human and a robot [18].

In this study, we focused on the latter approach, increasing the number of targets, because several studies showed the effectiveness of multiple robots that allocated rewards for improved performances [19,20] as well as in information-providing tasks [21–23] compared to a single robot. These studies concluded that participants preferred multiple robots over a single robot, even though the amount of information provided in these tasks was identical between multiple and single robots. Therefore, we pondered whether the number of such effects might occur in the context of *kawaii* feelings.

In considering the effect of the target number on feelings of *kawaii*, we must focus on two perspectives: the relationships between them and the total number. For the former, we focused on a sense of “communal sharing,” defined as a mental representation of a social relation, such as friendship [24]. A past study reported that affiliative contact between animals increases a sense of communal sharing, and high communal sharing videos are judged as cuter than low communal sharing videos [25]. A baby schema can effectively enhance the perceived *kawaii* feelings of the robots; similar to such a concept, we wondered whether showing a social relation between robots might raise the viewer’s feeling of *kawaii* toward them, including animals’ social relations.

Concerning the total number of targets, past studies reported that the number of robots did not linearly increase their social impact in the context of peer pressure [26]. Although this past work focused on peer pressure, which is a different kind of social influence by robots from our research topic, perhaps feelings of *kawaii* might also not increase linearly based on the number of robots. Although other past studies suggested the potential of the number of targets for enhancing feelings of *kawaii* [24,25], the total number and their mutual relationship effects have been inadequately investigated. Therefore, in this study, we investigate the relationships among the total number of targets, their mutual relationship, and perceptions of *kawaii*.

## Related work and research questions

### Kawaii feelings in human-robot interaction

To achieve socially acceptable robots in daily environments, robotics researchers have investigated what kinds of factors are essential in various fields [27,28] and reported the importance of trust [29], useful functionality for assisting seniors [30] and childcare [31], and the effects of cuteness [32,33]. In the context of the latter and the *kawaii* feeling of robots, researchers focused on applying to the appearance design of robots [34–36] the baby schema concept, which effectively raised the perceived feelings of *kawaii*. In fact, recent commercial products in social robot categories are designed to have cute appearances [1–3].

From another perspective, researchers investigated what behaviors can increase feelings of *kawaii*. For example, researchers reported the effectiveness of increasing *kawaii* feelings by a locomotion behavior design for a mobile robot [17] and a touch behavior for an information-providing robot [18]. These studies showed the usefulness of behavior design for improving feelings of *kawaii*.

Unfortunately, although these studies described the importance of *kawaii* feelings and approaches for raising such feelings, they only concentrated on interaction situations with a single robot. Even though recent social robot studies unveiled the usefulness of using multiple robots from various perspectives (see next subsection), the effects of their numbers and relationships toward *kawaii* feelings remain relatively unknown.

### Number effects in human-robot interaction

Using multiple robots simultaneously for a single application, such as conversational-based information-providing tasks, is one recent trend toward increasing their performances. For example, researchers showed that using multiple robots in conversation enables them to compensate for speech recognition failures by controlling conversational flow between robots, regardless of user answers [22,37]. Other studies described increases in the social impact of using multiple robots compared to a single robot, e.g., greater social reward effects [38], higher children’s motivations [23], and behavior changes due to peer pressures from multiple robots [26,39].

Although these studies investigated the effects of the number of robots on their social influences, they focused less on the relationships between the number of robots and feelings of *kawaii*. Therefore, it is unknown whether people perceived more *kawaii* feelings toward multiple robots than a single robot. However, the relationship between the number of robots and social influences might not be linear because another study concluded that the peer pressure effects from robots are not simply increased by adding more robots [26].

We also need to focus on the relationship effects among robots to understand the effects of multiple robots. Another past study reported the importance of a sense of communal sharing, induced by observing positive social relationships of humans or animals [25]. Another study argued that multiple robots literally touching each other, i.e., showing more affiliative relationships, can effectively provide information to people [40], suggesting the usefulness of showing positive relationships even among robots.

### Research questions and overview of the experiments

As summarized above, the number of robots and displays of their mutual relationship (e.g., friendliness among robots) might enhance feelings of *kawaii*. However, such numbers and relationship effects have not been investigated in the context of *kawaii*. Based on these considerations, we address the following question:

#### Research Question (RQ1)

Does observing two targets that display a mutual relationship enhance feelings of *kawaii* toward them?

Related to RQ1, a past study reported that people describe their feelings as being cuter when they observe high communal sharing videos (e.g., young animals that show affiliative contacts) rather than low communal sharing videos [25]. This phenomenon suggests that the number of items and their relationships influences feelings of *kawaii*. Perceptions of a social relation may not be limited to humans and animals because people easily anthropomorphize objects [41,42]. Therefore, we hypothesized that displaying a mutual relationship will increase the perceived feeling of *kawaii*, regardless of the targets’ characteristics.

#### Hypothesis 1 (H1)

People will experience greater feelings of *kawaii* toward targets that display their mutual relationship than targets that do not display such a relationship or a single target, regardless of the kinds of targets.

We conducted two experiments to answer RQ1 and H1. We first conducted a feasibility study that employed static images of different targets: human beings (twin boys/girls), non-human objects (cherries), and social robots. We employed these different targets to investigate the number and the mutual relationship effects that occur regardless of the target’s characteristics. Next, we conducted a follow-up experiment to investigate whether the same effects are observed for videos of two robots. Here we only used robots as video stimuli because our primary interest was in the behavior design of social robots.

If the results of these studies reveal the effectiveness of the number of robots and display their mutual relationship, a new research question emerges: what is the optimal number of robots that induces the strongest feeling of *kawaii*? We note that this additional research question is influenced by the answer to this research question, which remains unresolved. Moreover, as described above, few studies have examined the effect of the number of robots, complicating the creation of adequate hypotheses or predictions regarding their effective number. Therefore, in this study, we set the following exploratory research question.

#### Exploratory Research Question (RQ2)

How many targets induce the strongest feeling of *kawaii*?

To answer it, we conducted a third experiment with multiple robots to investigate the number of effects on feelings of *kawaii*.

## Experiment 1

### Materials and methods

All the procedures were approved by the Advanced Telecommunication Research Review Boards (21-501-3).

#### Visual stimuli and conditions

We conducted a web survey with photos of four targets: twin boys, twin girls, cherries, and robots. Thus, we used four levels in the *model* factor (boy, girl, cherry, and robot). We used royalty-free materials from https://www.photo-ac.com/ for the twin boys (model release obtained) and cherries and from https://www.pexels.com/ for the twin girls (model release obtained). We used a commercial robot, *Sota* (VSTONE, Japan), for the robot photos. It has eight degrees of freedom (DOFs): three for its head, two for each arm, and one for its lower body. Its height is 28 cm. Sota has a microphone, a camera, and a voice synthesis function to autonomously interact with people.

**Fig 1:**
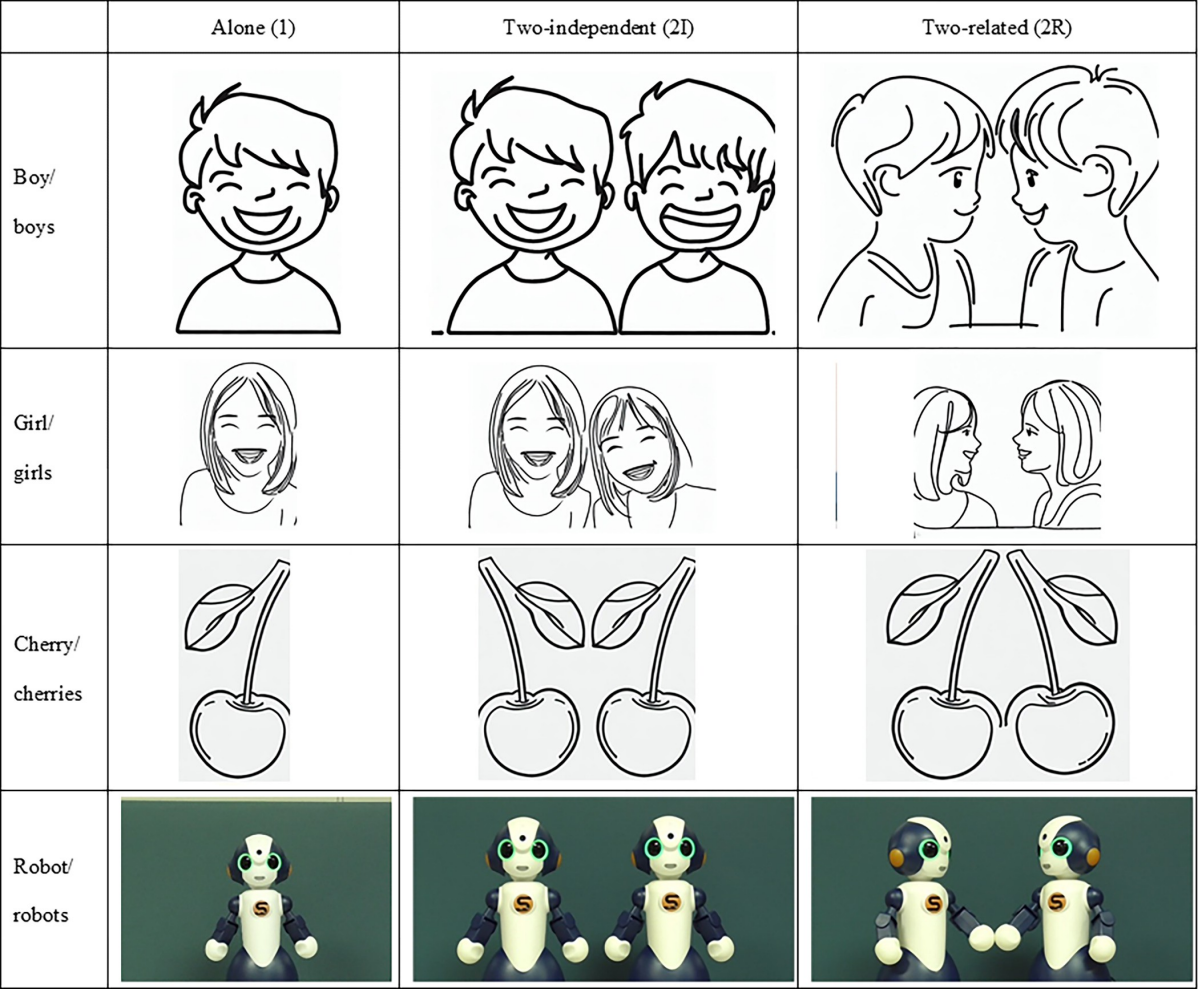
Photo images in Experiment 1. The images of boys, girls, and cherries are replaced with illustrations. The URLs for the original photo images are available in the appendix.

We used three levels in the *relationship* factor: one target (*alone* condition), two targets that did not display any mutual relationship (*two-independent* condition), and two targets that did display their mutual relationship by looking at and/or touching each other (*two-related* condition). Three photos for each target are shown in Fig 1. For the *two-related* conditions, the two targets looked at each other (twins and robots) and/or made physical contact (cherries and robots) to display their mutual relationship. In total. We prepared 4 x 3 pictures for four model conditions (boy, girl, cherry, and robot) and three relationship conditions (alone, two-independent, and two-related).

#### Measurement

We used a single questionnaire item to investigate the strength of the feelings of *kawaii*. This question has a strong positive correlation with degrees of wanting-to-approach [9,18], pleasure [9], and viewing durations in a free-viewing task [9], at least for Japanese participants whom we targeted in the present study. The item (“the degree of the feeling of *kawaii”*) was assessed on a 0-to-10 response format, where 0 meant “*not kawaii at all*” and 10 meant “extremely *kawaii*.” We employed an 11-point response format because a previous paper argued that this style more closely approximates interval data [43].

#### Procedure

First, the participants read explanations of the experiment and how to evaluate each photo. Next they observed all of the photos of the targets with the *alone*, *two-independent*, or *two-related* conditions and completed questionnaires for each photo. We employed a within-participant design where the participants observed 4 x 3 (12) pictures and evaluated them. The order of the photos and the types of targets were counterbalanced. Finally, they answered dummy questions to verify the quality of their answers because past research reported the need for such screening of participants in web surveys [44,45]. To detect lazy and dishonest participants, we prepared three dummy items using an example instruction manipulation check from previous work [45]. The text’s conclusion in the instructions explicitly asked participants to skip the answers on that page. We excluded participants who answered them.

#### Participants

Our experiment was conducted using participant pools of a Japanese survey company. A total of 201 people joined it: 99 females, 101 males, and 1 who declined to specify gender. Their average age was 41.74 years old (standard deviation (S.D.) was 9.67, and their age range was from 20s to 60s). The screening process, i.e., dummy questions as described above, winnowed that number to 162 valid participants: 77 women, 84 men, and 1 who declined to specify gender. Their average age was 41.80 years old (S.D. was 9.71). Participants gave written consent through an online consent form in the beginning, and they could withdraw from the study at any time.

### Results

**Fig 2:**
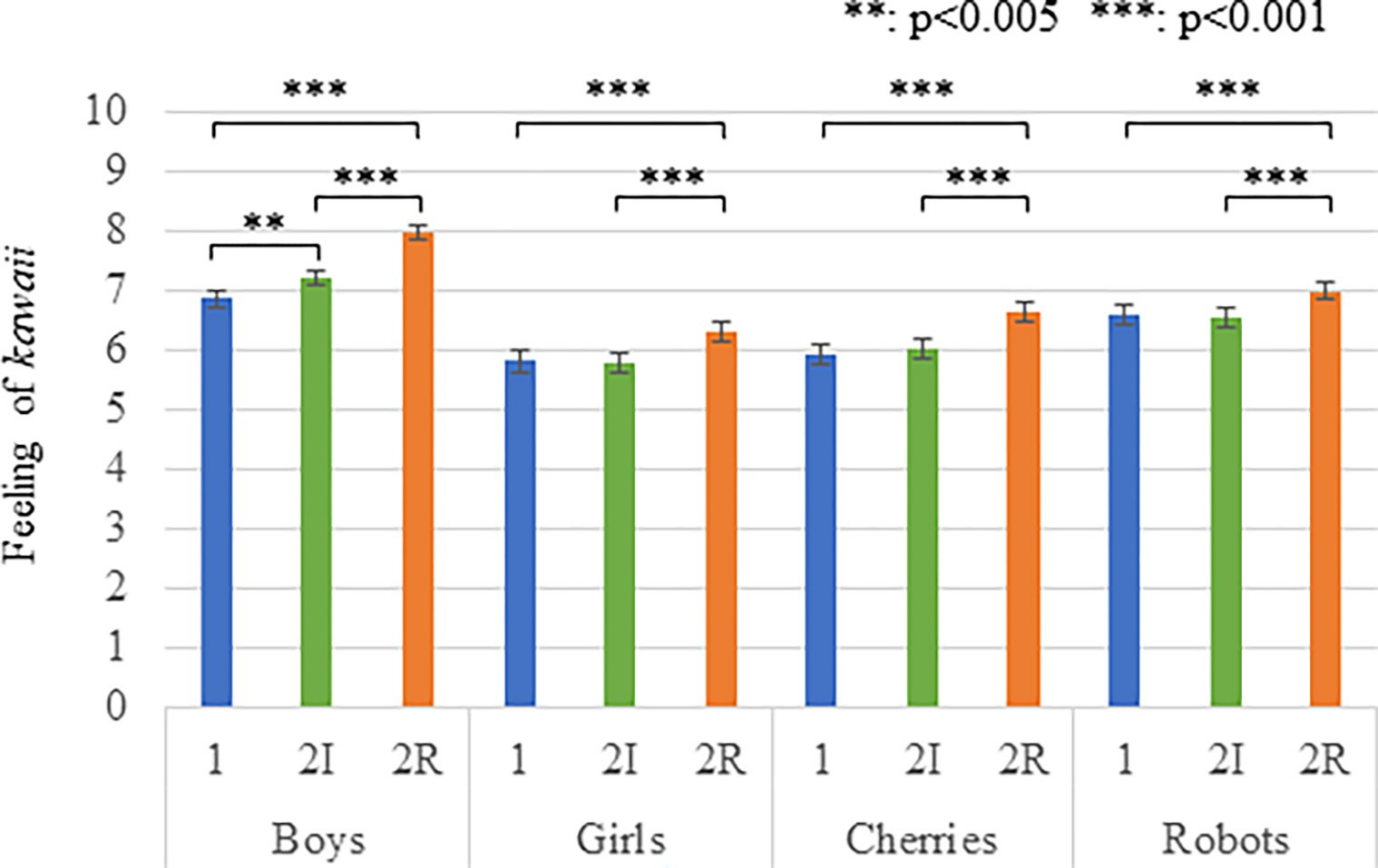
The feelings of *kawaii* measurements of Experiment 1. Means and standard errors (S.E.) of questionnaire rating scores about feelings of *kawaii* are shown (1: alone condition, 2I; two-independent condition, 2R: two-related condition).

Fig 2 and Table 1 show the mean and standard error (S.E.) of the rating scores for the photos. Although the Kolmogorov-Smirnov test showed that the feelings of *kawaii* measurements were not normally distributed, skewness and kurtosis values between -2 and +2 are considered acceptable to prove normal univariate distribution [46,47], and these values are within the ranges in our results. Therefore, we conducted a two-factors (relationship and model) repeated measures ANOVA for the degree of *kawaii* and found significant differences in the *relationship* factor (*F*(2,966) = 65.983, *p <* 0.001, *partial η2* = 0.291), in the *model* factor (*F*(3,966) = 33.428, *p <* 0.001, *partial η2* = 0.172), and in the interaction effects (*F*(6,966) = 5.856, *p <* 0.001, *partial η2* = 0.035).

**Table 1:** The mean and S.E. of questionnaire rating scores about feelings of *kawaii* of Experiment I.

	Alone	Two-independent	Two-related
Boy/boys	6.858 (0.150)	7.210 (0.149)	7.981 (0.132)
Girl/girls	5.920 (0.150)	6.025 (0.149)	6.636 (0.153)
Cherry/cherries	5.815 (0.179)	5.778 (0.170)	6.302 (0.174)
Robot/robots	6.580 (0.165)	6.537 (0.176)	6.994 (0.150)

Multiple comparisons with the Bonferroni method showed significant differences in the *boy/boys*: *alone < two-related* (*p* < 0.001), *two-independent < two-related* (*p* < 0.001), *alone < two-independent* (*p =* 0.001), *girl/girls*: *alone < two-related* (*p* < 0.001), *two-independent < two-related* (*p* < 0.001), *Cherry/cherries*: *alone < two-related* (*p* < 0.001), *two-independent < two-related* (*p* < 0.001), and *robot/robots*: *alone < two-related* (*p* < 0.001), *two-independent < two-related* (*p* < 0.001).

The experiment results showed that participants felt a greater sense of *kawaii* toward the targets that displayed their mutual relationship than targets that did not display such a relationship or a single target Thus, H1 is supported.

### Discussion

Our experiment results showed that the participants had more *kawaii* feelings during the *two-related* conditions than for the *two-independent* and *alone* conditions for all types of targets. On the other hand, the relationship between the *two-independent* and *alone* conditions did not show any significant differences except for the two boys. One possible reason for this difference between human and robot targets is based on very few differences in appearances between targets. A past study reported that clone images of humans induce negative feelings [48]. Our study used images of twins who are similar but not identical to each other in terms of facial expressions and poses. In contrast, the robots’ appearances are basically the same because they are consumer products. Multiple clone robots might induce negative feelings. Nevertheless, our experimental results suggest that feelings of *kawaii* can be increased by displaying a mutual relationship shared by the targets, even if their appearances are identical.

## Experiment 2

Experiment 2 investigated the effects of the number of robots and their relationship using video clips as an additional investigation related to H1.

### Materials and methods

All the procedures were approved by the Advanced Telecommunication Research Review Boards (21-501-3).

#### Visual stimuli and conditions

**Fig 3:**
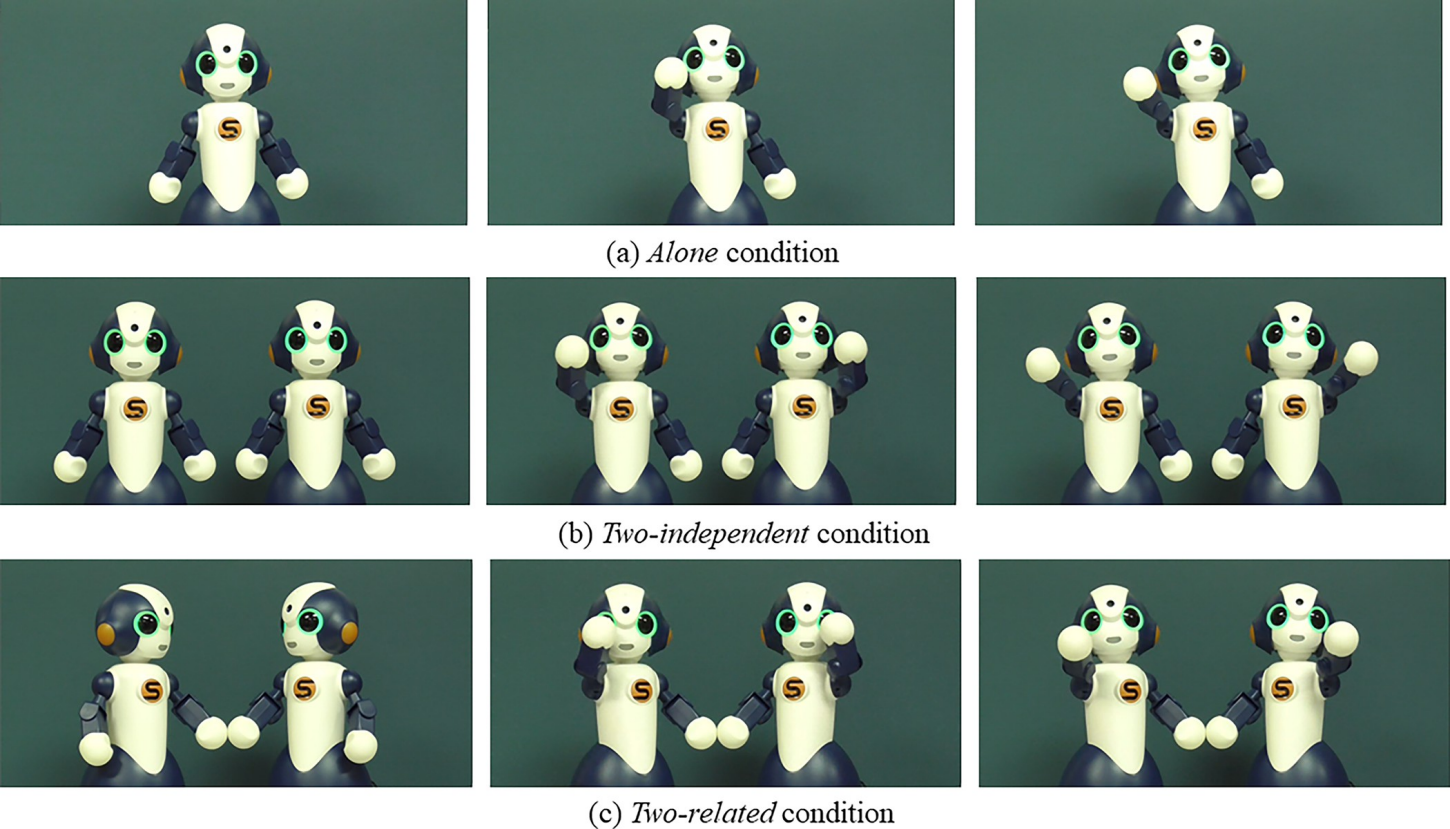
Video stimuli used in Experiment 2. Snapshots of videos are shown here.

We prepared 8-second videos in which a robot (robots) waved its (their) hand and said “bye-bye” (goodbye) with/without displaying any mutual relationship. We again used *Sota*, which has an LED on its mouth that blinks to indicate the voice volume of the robot. Similarly to Experiment 1, we compared three conditions: *alone*, *two-independent*, and *two-related*. We prepared three videos for Experiment 2. Each video’s resolution was 1920 × 1080 pixels, with 30 frames per second. Fig 3 shows screenshots from each condition. In the *alone* condition, a robot looks to the front, waves its right hand twice, and says “bye-bye.” In the *two-independent* condition, two robots look to the front, wave their outside hands, and say “bye-bye.” In the *two-related* condition, to display their mutual relationship, we employed eye-contact and physical-contact behaviors: two robots held hands from the beginning, made eye contact, waved their outside hands, and said “bye-bye.” The video clips are available in the supplementary materials.

#### Measurement

We investigated the feelings of *kawaii* with the identical questionnaire item from Experiment 1: the feeling of *kawaii* (0 = *not at all*, 10 = *extremely*).

#### Procedure

All the procedures were approved by the Advanced Telecommunication Research Review Boards (21-501-3). First, the participants read explanations of the experiment and how to evaluate each video, and then we verified that they could clearly hear the audio. Next they observed a video with *alone*, *two-independent*, or *two-related* conditions and completed questionnaires for each one. Therefore, we also employed a within-participant design for this experiment. Our participants observed and evaluated three videos whose orders were counterbalanced.

#### Participants

Our experiment was conducted using different participant pools of a Japanese survey company, i.e., no participants from Experiment 1. A total of 202 people joined it: 100 females, 100 males, and 2 who declined to specify gender. Their average age was 41.64 years old (S.D. was 9.79, and their age range was from 20s to 60s). The screening process winnowed that number to 179 valid participants: 87 women, 90 men, and 2 who declined to specify gender. Their average age was 41.44 years old (S.D. was 9.65). These participants were not involved in the first experiment.

### Results

**Fig 4:**
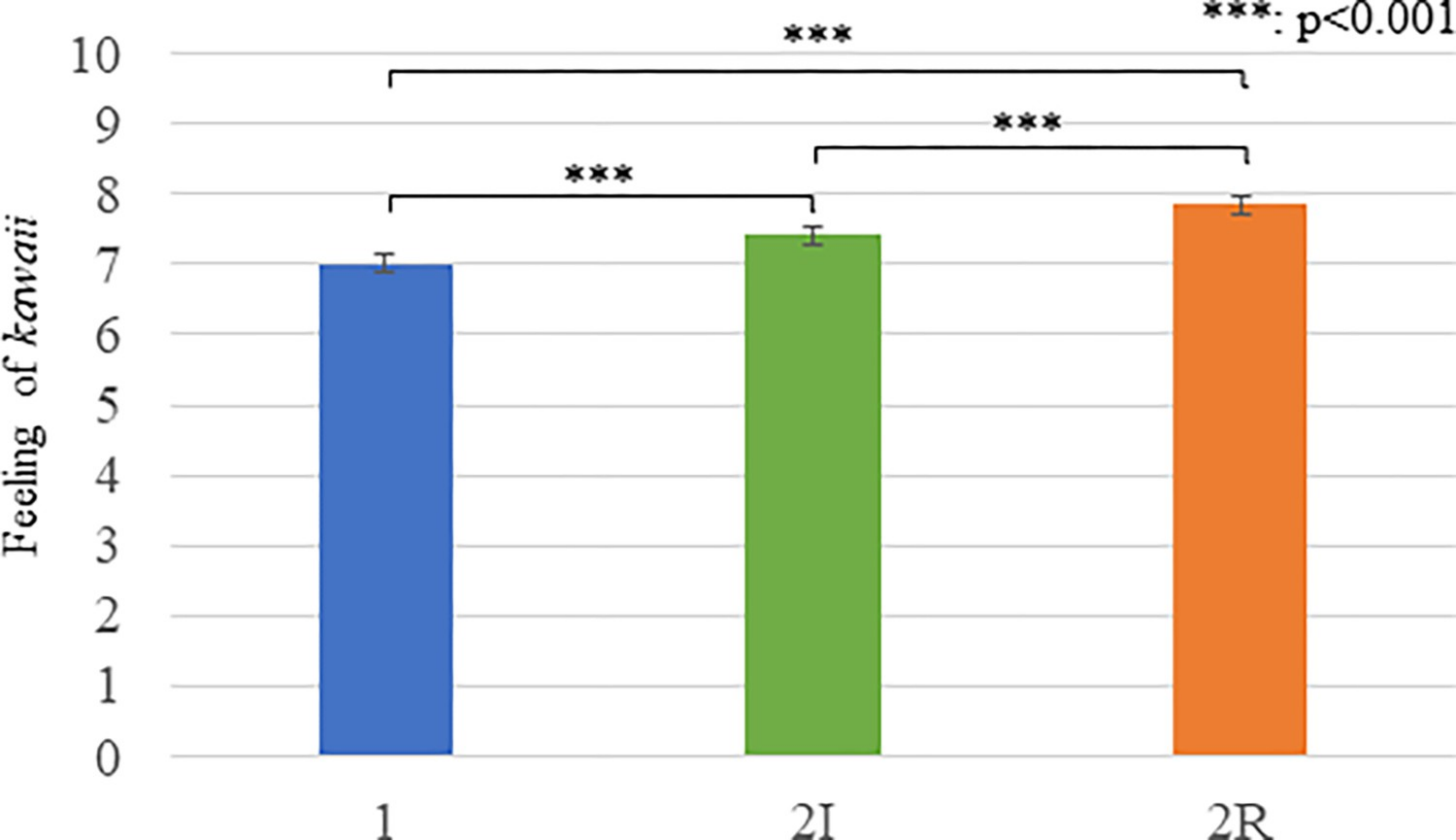
The feelings of kawaii measurements of Experiment 2. Means and standard errors of questionnaire scores about feelings of kawaii are shown (1: alone condition, 2I; two-independent condition, 2R: two-related condition).

The Kolmogorov-Smirnov test showed that the feelings of *kawaii* measurements were not normally distributed, and the skewness and kurtosis values were outside the range between -2 and +2. Therefore, we conducted a Friedman test for the *kawaii* scores and identified a significant difference (*X*^*2*^(2) = 82.657, *p <* 0.001). As shown in Fig 4 and Table 2, Multiple comparisons found significant differences between conditions: *alone* < *two-related* (*p* < 0.001), *two-independent* < *two-related* (*p <* 0.001), and *alone < two-independent* (*p <* 0.001). These experimental results showed that the participant felt a stronger sense of *kawaii* toward the robots that displayed their mutual relationship than those that did not display such relationships or a single robot, even though they are not static images. The results also provide additional evidence that supports H1.

**Table 2:** The mean and S. E. of questionnaire rating scores about feelings of *kawaii* of Experiment 2.

	Alone	Two-independent	Two-related
Robot/robots	7.006 (0.126)	7.419 (0.133)	7.855 (0.127)

### Discussion

Experiment 2 showed that our participants experienced more feelings of *kawaii* toward two robots that displayed their mutual relationship than toward two robots that did not display such a relationship or a single robot. However, unlike Experiment 1 in which two-independent robots did not significantly outperform a single robot, Experiment 2 showed their advantages. A possible reason for this discrepancy may be that the robots in Experiment 2 moved line-symmetrically and gave fewer clone-like impressions than the static robots in Experiment 1.

These results also provide an implication for interaction design policy in robot-robot interactions. Although past studies reported the effectiveness of using multiple robots for information-providing tasks [21–23], they concentrated less on the effects of displaying mutual relationships among robots. Perhaps the robots in the previous studies were implicitly designed to interact with each other, and perceiving such interactions may make positive contributions. Our experiment results provide evidence for the merit of explicitly designing a “perceivable” interaction between robots when robot developers intend to use multiple robots for their services.

The results of Experiments 1 and 2 consistently show the advantage of multiple robots that display their mutual relationships over a single robot to enhance a viewer’s feeling of *kawaii*. However, the comparison was limited to one or two robots.

## Experiment 3

Experiment 3 investigated the best number of robots for inducing the strongest feeling of *kawaii*. Because we investigated RQ2 in an exploratory manner, we did not prepare any predictions for it.

### Materials and methods

All the procedures were approved by the Advanced Telecommunication Research Review Boards (21-501-3).

#### Visual stimuli and conditions

**Fig 5:**
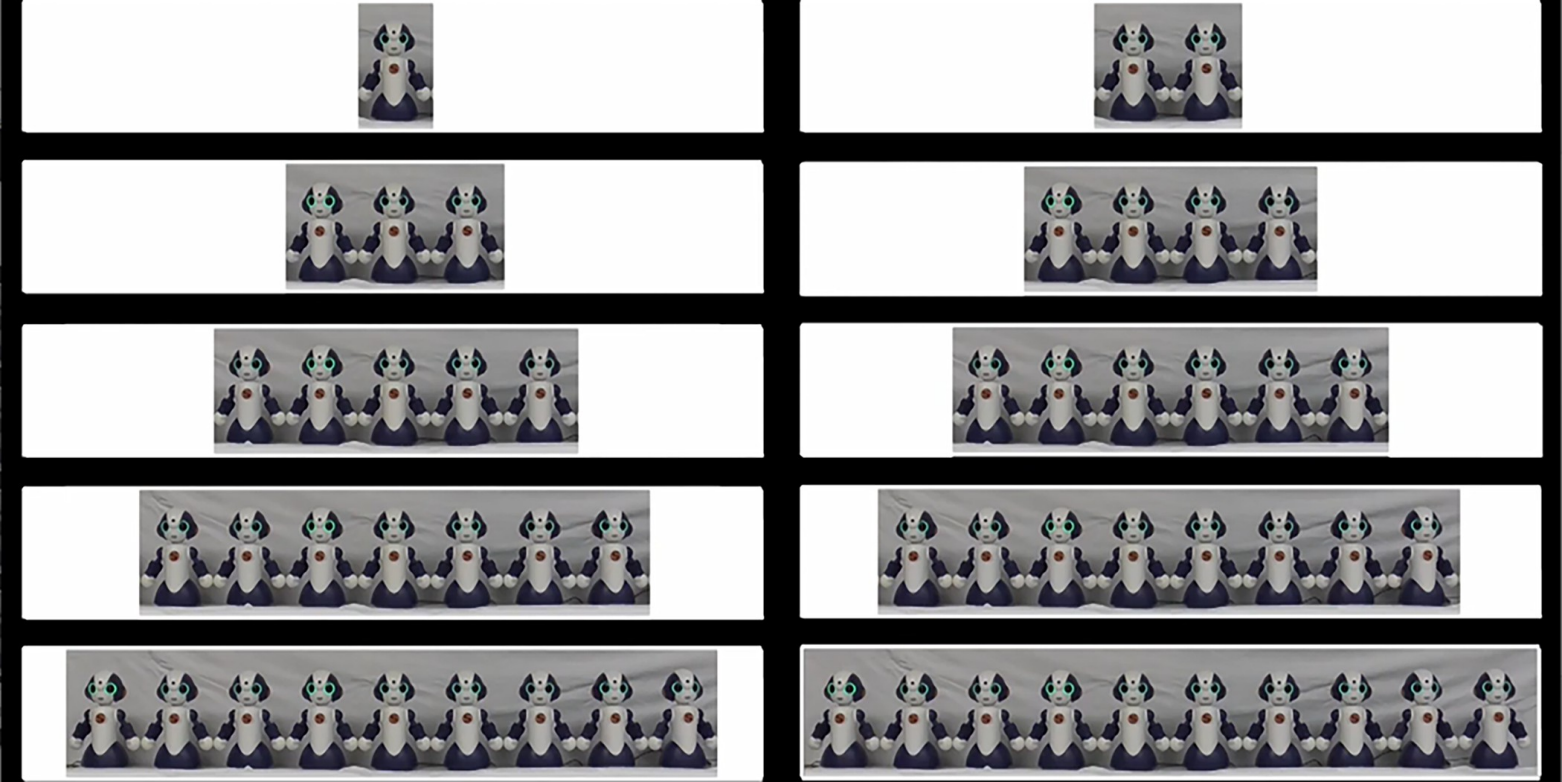
Video stimuli used in Experiment 3. Snapshots of ten videos are shown here.

**Fig 6:**
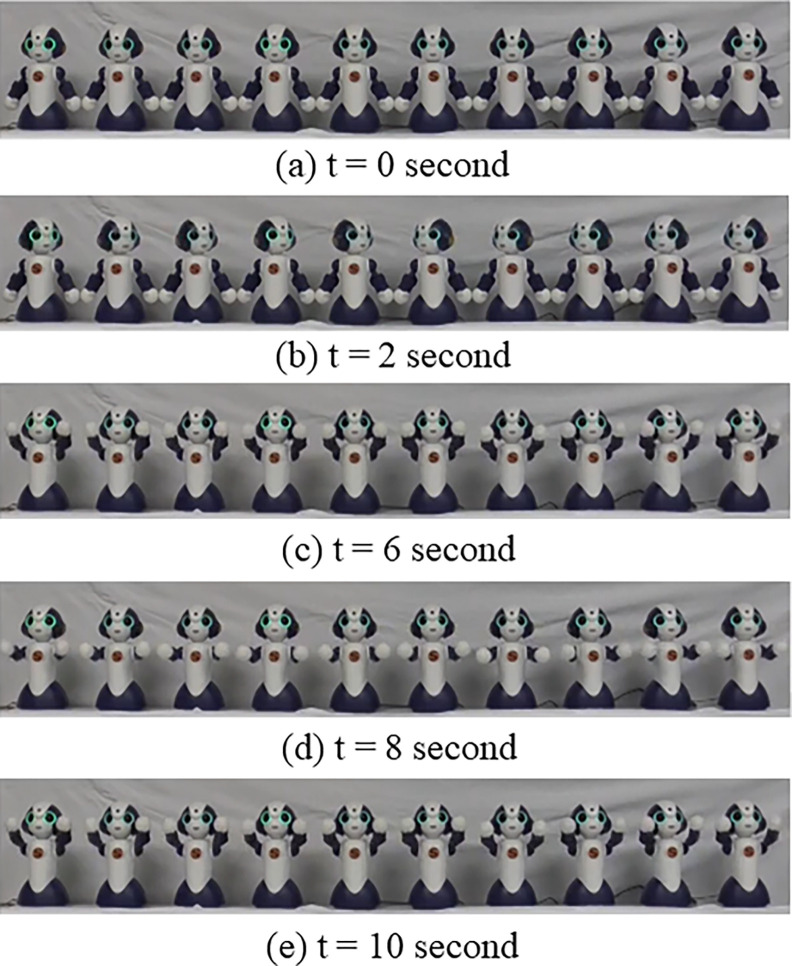
Time course of ten robots’ video used in Experiment 3. Snapshots of picture frames in ten-robot condition are shown here.

Based on the results of Experiments 1 and 2, we used similar behaviors for the robots. They held hands, made eye contact, nodded to adjust to the timing of the next action, and waved “goodbye.” We manipulated the number of robots from one to ten and took 12-second videos for each, i.e., this experiment has ten conditions: *1*, *2*, *3*,*4*, *5*, *6*, *7*, *8*, *9* and *10*. Fig 5 shows screenshots from the video stimuli. In the *1*condition, a robot looked straight ahead and waved its hands. In the *2* to *10*conditions, when the number of robots was even (Fig 6), the robots held hands, made eye contact with their opposite side partner, nodded to adjust the timing of the next action, and waved both hands. When the number of robots was odd, the robots behaved similarly, except that the robot in the center looked ahead and moved like the robot in the *1*condition. The robots used both hands while waving, which is different from Experiment 2. Video clips are available in the supplementary materials.

#### Measurement

We investigated the feelings of *kawaii* with the same questionnaire item from Experiments 1 and 2: feelings of *kawaii* (0 = *not at all*, 10 = *extremely*).

#### Procedure

All the procedures were approved by the Advanced Telecommunication Research Review Boards (21-501-3). First, the participants read explanations of the experiment and how to evaluate the video, and then we verified that they could clearly hear the audio. Next they observed the ten videos and answered questionnaires. The order of the ten videos was counterbalanced. They could repeat them as often as they liked while answering them. Finally, they answered dummy questions to verify the quality of their answers.

#### Participants

Experiment 3 was conducted using different participant pools from a Japanese survey company, i.e., no participants from Experiments 1 or 2 joined Experiment 3. 200 people joined Experiment 3: 98 women and 102 men whose average age was 41.4 years old (S. D. was 9.51, their age range was from 20s to 60s). The screening process winnowed that number to 152 valid participants: 67 women and 85 men whose average age was 41.43 years old (S. D. was 9.36).

### Results

**Fig 7:**
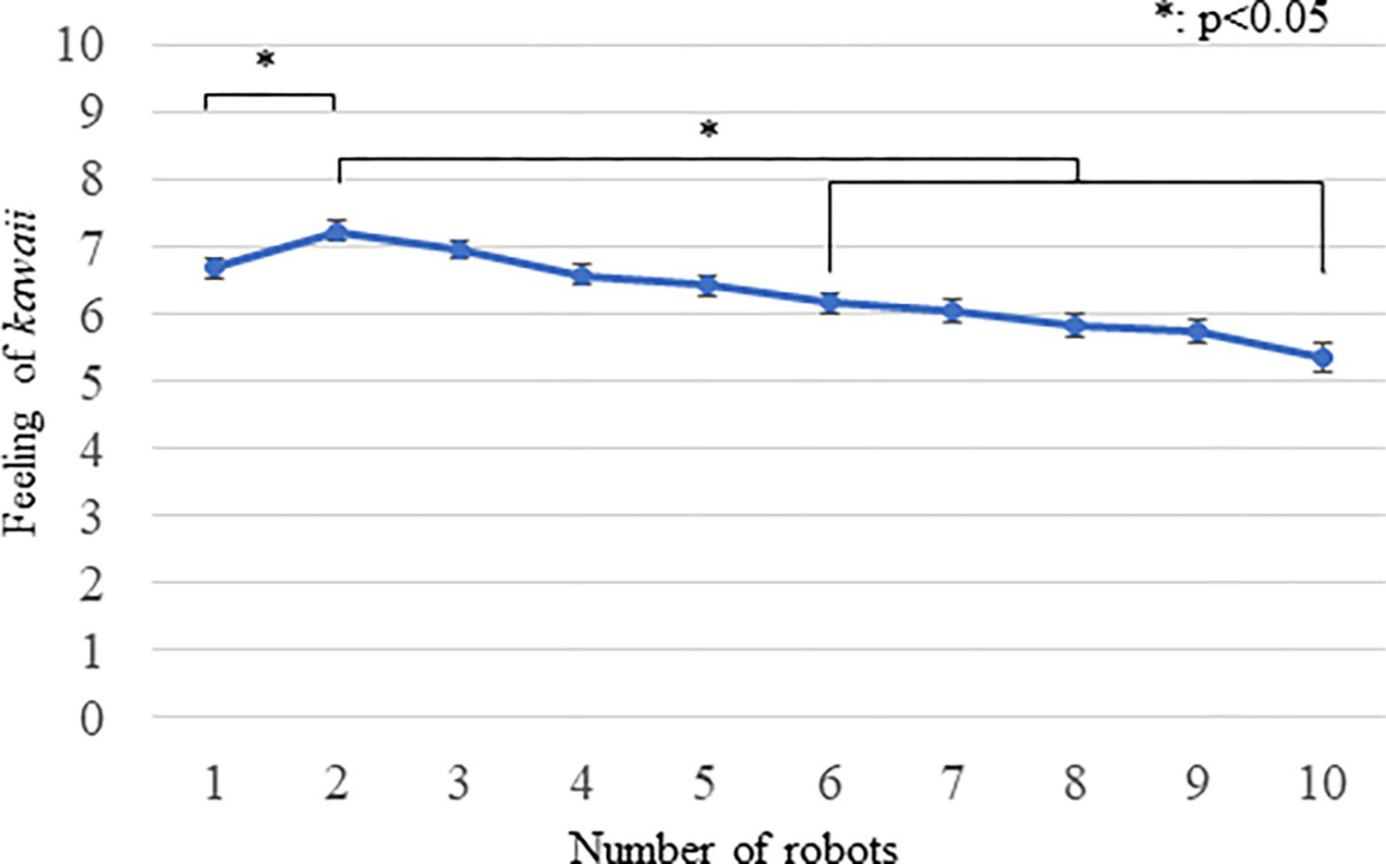
The feelings of kawaii measurements of Experiment 3. Means and standard errors of questionnaire scores about feelings of *kawaii* are shown. We only illustrated significant differences between *2* and other numbers to avoid complications.

Fig 7 and Table 3 show the mean and S.E. of the ratings. Because the Kolmogorov-Smirnov test showed that the feelings of *kawaii* measurements were not normally distributed and skewness and kurtosis values were outside the range between -2 and +2, we conducted a Friedman test for the *kawaii* scores. The analysis showed a significant difference (*X*^*2*^(9) = 169.084, *p <* 0.001). Multiple comparisons with the Bonferroni method found significant differences (all *p* values are < 0.05) between conditions: *2* > [*1*, *6*, *7*, *8*, *9*, *10*], *3* > [*6*, *7*, *8*, *9*, *10*], *4* > [*8*, *9*, *10*], *5* > [*8*, *9*, *10*], *1* > [*9*, *10*], *6* > *10*, and *7* > *10*. These results showed the advantages of *two* robots in the context of perceiving *kawaii* feelings compared to six or more robots. Although statistical comparisons among two to five robots are not significant, only two robots are cuter than one robot within these numbers.

**Table 3:** The mean and S. E. of questionnaire rating scores about feelings of *kawaii* of Experiment 3.

	One	Two	Three	Four	Five
Robot/robots	6.671 (0.137)	7.230 (0.139)	6.941 (0.141)	6.566 (0.146)	6.421 (0.144)
	Six	Seven	Eight	Nine	Ten
Robot/robots	6.158 (0.155)	6.033 (0.170)	5.822 (0.189)	5.737 (0.193)	5.349 (0.209)

### Discussion

Experiment 3 again showed that participants experienced more feelings of *kawaii* toward two robots that displayed their mutual relationship than a single robot, suggesting that two robots are better choices for increasing feelings of *kawaii* when the robots’ appearances are identical. Therefore, the results answered RQ2; to increase the feeling of *kawaii* using targets that have the same appearance, two robots are appropriate choices based on these measurements.

This result also highlights an implication for interaction design policy in the representations of the behaviors of multiple robots. Similar to Experiment 2, we provide evidence for the merit of using a small number of multiple robots to increase feelings of *kawaii*. Robotic companies often use a large number of robots to demonstrate their advanced technology to control them synchronously. Although such an appeal emphasizes the accuracy of their systems, it will not increase the degree of *kawaii* of their products.

One interesting phenomenon is that the participants experienced similar *kawaii* feelings for one and four robots. The number four is one threshold for possible precise counting without language [49–51]. Therefore, these results might be related to the limitation of number perception in humans.

One limitation of this study is the formation of the robots. We only investigated feelings of *kawaii* when they were lined up side by side. However, if their formations were different, perhaps triangular, the perceived *kawaii* feeling might be changed even when the number of robots is relatively large. Such various formations might create different perceived relationships among robots, such as leaders and followers, which would also undoubtedly influence the perceived feelings. If many robots are crowded together, people observing such a scene might perceive mutual relationships and have positive impressions. Investigating the connection between the formation and perceived mutual relationships is an interesting future work based on our study.

## General discussion

Our present three experiments consistently show that the number of targets and their perceived relationship affect the feelings of *kawaii* toward them. Our participants evaluated a small number of multiple targets that displayed their mutual relationship as most *kawaii*. This phenomenon resembles a previous finding where a sense of communal sharing between humans/animals enhanced cuteness perceptions [25] and suggests that people can also experience communal sharing feelings from artificial agents like robots. This knowledge is useful for behavior designs for robot-robot interaction. It might also be transferable to other kinds of consumer products than robots, for instance, in shopping environments. For example, even when using such immobile toys as stuffed animals or cartoon character merchandise, changing their postures and positions to imply a mutual relationship could increase *kawaii* feelings in customers.

Most past studies investigating the effects of multiple robots used a maximum of two robots [19–23]. The number of suitable robots might depend on their applications and services, although our exploratory research approach provides evidence for the advantages of using two robots and showing their mutual relationships. The number of effects has been investigated with social robots and CG-based agents [19]. Therefore, our knowledge can be used for designing such agent-based interactions.

One typical research topic that uses more than two robots is peer pressure. Researchers used more than two robots in studies in the context of peer pressure effects from robots [39,52,53]. They investigated the confirmation effects of multiple robots with four or six robots and concluded that two are not enough [26]. However, in these studies, the robots behaved in relation to each other like strangers. Therefore, if they exhibited mutual relationships in advance, their social influence might be changed even when the number of robots is two.

This study has several limitations regarding the appearance and behaviors of its visual stimuli. We only used images of specific targets (i.e., twins, cherries, and robots). Testing with different types of objects is critical to examine how closely the current knowledge is applicable to various targets. For example, more human-like robots like androids [54–56] or cuter pet-like robots [3,57] might induce different *kawaii* feelings. In addition, the number of targets with different appearances (e.g., not twins, different objects, and robots) and displaying various relationships (e.g., a leader and followers) will influence feelings of *kawaii* differently. The robots’ motions were also limited to a simple greeting behavior whose motion timing was synchronized. Since such synchronized robot motions influence people’s perceptions [52], if the timing of robot motions were not synchronized, observer impressions would undoubtedly change.

Despite these limitations, our results still provide basic knowledge about investigating the relationship between the number of targets and feelings of *kawaii*. For example, if robotics researchers investigate the effects of synchronized behavior among multiple robots with different appearances on the feelings of *kawaii*, a comparison with our results will provide interesting knowledge for a deeper understanding of the relationship among number, appearance, and behavior synchronizations. Moreover, using robots with an identical appearance was a realistic approach in past studies on the effects of multiple robots; therefore, the knowledge from our study also provides a useful design guideline for researchers who are tackling this research topic [19–23].

One possible future work is to investigate the number and relationship effects with different languages and cultures. Although awareness of the word *kawaii* is spreading worldwide, this emotion has been expressed differently in other languages, such as cute in English. Of course, cute is not exactly the same expression as *kawaii* in Japanese [4,5]. Another study investigated whether Spanish has a word equivalent to *kawaii* [58] and reported that *tierno/tierna* was used similarly to *kawaii* in Japanese and *cute* in English to describe human and baby animals, although *kawaii* had broader meanings than both words. In addition, an emotion evoked by seeing something cute is called “elérzékenyült in Hungarian, heldinud in Estonian, heltyä in Finnish” [35]. This study argued that cuteness evokes *kama muta*, a social-relational emotion. Therefore, perhaps different languages and cultures might influence the relationships between the number of robots and feelings of *kawaii*. Understanding such differences may provide interesting knowledge and guidelines for designing multiple robot behaviors in global markets.

## Conclusion

The present study is the first to investigate the effects of the number of targets and their perceived relationships on viewer’s feelings of *kawaii*. Experiment 1 was conducted with static images of three different types of targets. Displaying a mutual relationship between two targets was felt to be more *kawaii* than a single target or two targets that did not display any mutual relationship, regardless of their types. Experiment 2 was conducted with videos in which one or two robots simply waved, and two robots that displayed their mutual relationship were most highly evaluated, similar to Experiment 1.

In Experiment 3, we exploratorily investigated the best number of robots to induce feelings of *kawaii* and found the advantages of using two robots. Despite its exploratory nature, this study advances knowledge about the eliciting conditions of *kawaii* feelings. Both the physical appearance of a single target and its number and perceived social relationships are related to feelings of *kawaii*.

## Supporting information

S1 File Anonymized data set. (XLSX)

S2 File Visual stimuli. (ZIP)
